# Use of *Brucella abortus* species specific polymerase chain reaction assay for the diagnosis of bovine brucellosis

**DOI:** 10.4102/jsava.v88i0.1433

**Published:** 2017-09-27

**Authors:** Songelwayo L. Chisi, Tracy Schmidt, George W. Akol, Henriette van Heerden

**Affiliations:** 1 Department of Agriculture and Rural Development KwaZulu Natal Province, Allerton Provincial Veterinary Laboratory, South Africa; 2 Centre of Veterinary Excellence, Dohne Agricultural Development Institute, South Africa; 3 Department of Rural Development and Agrarian Reform, Stutterheim, South Africa; 4 Department of Tropical Diseases, University of Pretoria, South Africa

## Abstract

Serology is primarily used in the diagnosis of bovine brucellosis. Bacterial culture and isolation is the gold standard in diagnosing brucellosis but, like serology, it does not offer complete (100%) diagnostic sensitivity and specificity. Polymerase chain reaction (PCR) has been suggested to offer better specificity and sensitivity. In this study, we evaluated the performance of *Brucella abortus* species specific (BaSS) PCR directly from different samples in the diagnosis of bovine brucellosis in naturally infected cattle in KwaZulu-Natal province of South Africa with known infectious status from culture. The BaSS PCR had a low diagnostic sensitivity (DSe) of 70%, but was able to identify vaccine strains using abomasal fluid from aborted foetuses and detect *Brucella* DNA from decomposing samples. The best sample for the BaSS PCR was abomasal fluid.

## Introduction

Bovine brucellosis is a disease of cattle usually caused by *Brucella abortus* (Bishop, Bosman & Herr 1994). *Brucella abortus* biovar (bv) 1 causes 90% of infections in cattle, while 10% are because of biovar 2 in South Africa (Bishop et al. 1994). Control of bovine brucellosis in South Africa is achieved mostly by vaccination with *B. abortus* S19 of heifers between 3 and 9 months and RB51 vaccines, correct identification of positive reactors and removal and slaughter of positive reactors under the supervision of a state veterinarian.

## Communication

Bacterial culture and identification is the gold standard for the diagnosis of bovine brucellosis (Nielsen 2002). However, in some cases, culture yields negative results or seems impractical with large herds and huge numbers of animals (Office International des Epizooties [OIE] 2012). Serological tests therefore offer a more practical means of diagnosing brucellosis. However, more than one test is used to confirm bovine brucellosis because no single test absolutely identifies infection with *Brucella.*

*Brucella* specific polymerase chain reaction (PCR) assays using various samples including blood (Ilhan et al. 2008; Leal-Klevezas, López-Merino & Martínez-Soriano 1995), milk (Leal-Klevezas et al. 1995), tissue (O’Leary, Sheahan & Sweeney 2006) as well as stomach content and aborted foetuses (Cetinkaya et al. 1999; Cortez et al. 2001) have been reported. However, PCR has sensitivity problems because the stage of infection influences the number and location of *Brucella* organisms in white blood cells and lymphoid tissue glands (O’Leary et al. 2006). The *B. abortus* species specific (BaSS) PCR assay identifies and discriminates *B. abortus* field strain (wild-type biovars 1, 2 and 4), vaccine strains (S 19 and RB51), as well as other *Brucella* species and non-*Brucella* bacteria (Bricker et al. 2003).

In this study, *Brucella* status was determined by bacterial culture and isolation of samples from milk (*n* = 8), abomasal fluid from aborted foetuses (*n* = 35), uterine discharges (*n* = 2), hygroma fluid (*n* = 1) and lymph nodes (*n* = 2) submitted to Allerton Provincial Veterinary Laboratory (APVL) between 2009 and 2012 from seropositive cattle from commercial and communal herds located in KwaZulu-Natal province, South Africa. The lymph nodes were collected from abattoirs that slaughtered known brucellosis seropositive cattle, that is, seropositive animals with high (i.e. 392 or greater) complement fixation test (CFT) international units titres that were slaughtered under the supervision of a state veterinarian according to the law (Act 35 of 1984). The 48 samples were received from 28 farms with an abortion history in KwaZulu-Natal province. *Brucella* bacterial culture status of these samples as described by the OIE (2012) was performed at APVL and isolates were sent to the Agricultural Research Council-Onderstepoort Veterinary Institute (ARC-OVI) for identification at species level.

Of the 48 samples, 10 (21%) *Brucella* cultures were identified as *B. abortus* bv. 1 at ARC-OVI, South Africa. DNA was extracted from these samples (i.e. from abomasal fluid, milk, uterine discharges and lymph nodes) according to the procedure of a QIAGEN commercial kit (Qiagen GmbH, Qiagen strasse 1, 40724, Germany) and used as template in the BaSS-PCR assay as described by Bricker et al. (2003). In brief, the extraction was done as follows: 500 µL of abomasal fluid, milk, hygroma fluid or uterine discharge was placed in 1 mL Eppendorf tubes and 20 µL protease K and 180 µL ATL buffer were added to the mixture. The tubes were then placed in a heating block set at a temperature of 56 °C for 1 h. The tubes were periodically vortexed to ensure thorough mixing of the contents. The lysis was completed by placing 200 µL ATL in the mixture and vortexing. The DNA binding conditions were adjusted by placing 200 µL of ethanol to the mixture. The mixture was then added to the flow column (so that the DNA could bind to the column) and contents were centrifuged at 6000 g for 1 min. Then the silica membrane of the flow column was washed with 500 µL of AW1 and centrifuged at 6000 g for 1 min. Then AW2 was added and centrifuged at 17 200 g for 3 min. A dry spin at 6000 g for 1 min was done. To elute the DNA, 100 µL AE buffer was added to the column and the contents were left for 3 min at room temperature and then centrifuged at 6000 g for 1 min. Then 100 µL AE buffer was added and the contents were further centrifuged at 6000 g for 1 min. The liquid that was eluted and was now in the collecting Eppendorf tubes contained the DNA. In the case of lymph nodes, they were first macerated and mixed with 500 µL buffer and then followed the extraction process described above.

The concentration of the primers was 100X, which consisted of 50 µM 16S-F (5’GTG-CCA-GCA-GCC-GCC-GTA-ATA-C3’), 50 µM 16S-R (5’TGG-TGT-GAC-GGG-GGG-TGT-GTA-CAA-G3’), 50 µM of *B. abortus* specific (5’GAC-GAA-CGG-AAT-TTT-TCC-AAT-CCC3’), 50 µM RB51 (5’ GCC-AAC-CAA-CCC-AAA-TGC-TCA-CAA3’), 50 µM eriF (5’GCG-CCG-CGA-AGA-ACT-TAT-CAA3’), 50 µM eriR (5’CGC-CAT-GTT-AGC-GGC-GGT-GA3’) and 50 µM IS711 (5’TGC-CGA-TCA-CTT-AAG-GGC-CTT-CAT-TGC-CAG3’). Then the reaction mixture per one assay was prepared as follows: 5 µL PCR-grade water, 0.1 µL IS711, 0.1 µL *B. abortus* specific , 0.1 µL 16S-F , 0.1 µL 16S-R, 0.1 µL RB51, 0.1 µL eriF and 0.1 µL eriR. The master mix then consisted of 11.3 µL PCR-grade water, 2.5 µL 10X reaction buffer without MgCl_2,_ 0.25 Mm MgCl_2,_ 10 mM dNTPs, 2.5 µL reaction mixture from the above step, 5 µL PCR-grade water and 0.2 µL *Taq* DNA Polymerase (5 IU/uL; Promega). Then 24 uL of the master mix from the above composition and 1 µL of unknown sample was then aliquoted in PCR tubes and placed in a thermal cycler. The PCR process consisted of an initial denaturation cycle of 95 °C for 5 min (1 cycle), followed by 40 cycles of 95 °C for 15 s, 52 °C for 30 s and 72 °C for 90 s. The amplified samples were then electrophoresed in 2% agarose gel and 1X Tris-Borate-EDTA (TBE) buffer. Following electrophoresis, gels were stained in an ethodium bromide solution (10 mg/mL) and then visualised under ultraviolet (UV) light. A negative control and *B. abortus* as positive control (bacterial DNA) were also included.

**FIGURE 1:**
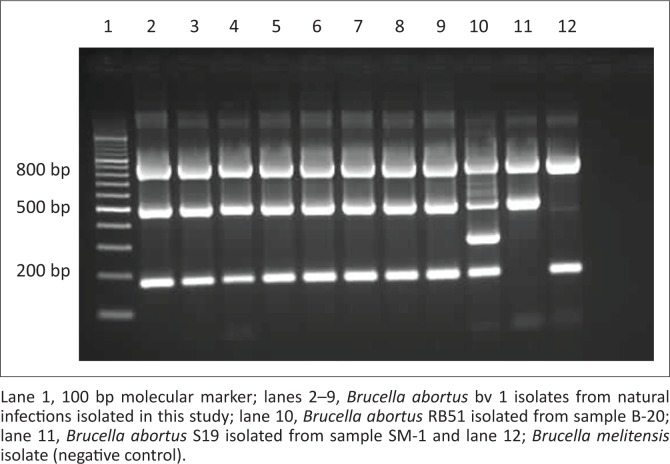
*Brucella abortus* species specific polymerase chain reaction products of samples collected from commercial and communal herds in KwaZulu-Natal in South Africa.

Amplification of a 500 bp *B. abortus* specific IS711 repeat element fragment in the *alk*B gene confirmed *B. abortus* infection. The 500 bp *B. abortus* specific IS711 repeat element fragment in *alk*B gene was detected in 7 of the 10 samples. The DSe was computed using results from 10 samples from which *B. abortus* was also simultaneously isolated; thus, the DSe of the BaSS was determined to be 70.0% (95% confidence interval [CI]: 39.0–94.0). The remaining 38 samples were also tested with the BaSS PCR and the results were as follows: no *Brucella* DNA was detected from the milk (*n* = 8), hygroma (*n* = 1), uterine discharges (*n* = 1) and abomasal fluid (*n* = 28). Two culture-negative abomasal fluid samples amplified a 350 bp fragment unique to *B. abortus* RB51 vaccine. The absence of 180 bp eri gene in one sample distinguished it as a *B. abortus* S19 (SM-1) vaccine strain (Bricker et al. 2003; Figure 1). *Brucella melitensis* and *B. abortus* (bacterial DNA) were included as controls. Serological tests detected the SM-1 animal as *Brucella* seropositive, while the BaSS PCR assay confirmed that animal SM-1 was vaccinated with S19. Also serological tests classified sera samples from two dams as sero-positive while the BaSS PCR detected *B. abortus* RB51 vaccine strain DNA from their aborted foetuses (B-20 and B-21). These abortions occurred shortly after the farmer vaccinated the herd with RB51 vaccine strain.

The DSe of the BaSS-PCR assay with known bacterial culture status was determined to be 70.0% (95% CI: 39.0–94.0) using 10 samples with known culture status. The DSe results are in agreement with previously reported DSe of 66.7% – 100% using cultures (Bricker et al. 2003). The BaSS PCR successively differentiated *B. abortus* wild strain from RB51 and S19 *B. abortus* strains. Differentiating vaccine strains from field infections is very important because of the drastic control measures that are imposed on farmers, that is, quarantine of farm and slaughter of affected animals. BaSS PCR lacks diagnostic sensitivity compared to serological tests but can be used effectively to confirm wild-type or vaccine-derived *B. abortus* during the waiting period for culture identification from abomasal fluid from aborted foetuses (within 2 days) or from decomposed samples.

## Conclusion

The BaSS PCR worked very well when abomasal fluid was the source of DNA. It is recommended that the BaSS PCR be considered when confirming *B. abortus* at species level as it can differentiate *B. abortus* wild strains from vaccine strains. It can be used during the waiting period for culture and identification as the BaSS PCR is able to identify *B. abortus* from abomasal fluid within 2 days compared to culture identification which can take up to 3 weeks.
